# Profiles of parental response, intersectional microaggressions, and mental health problems among sexual and gender minority youth of color

**DOI:** 10.1111/jora.70219

**Published:** 2026-06-22

**Authors:** Shixin Fang, Meg D. Bishop, Cara L. Exten, Hongjian Cao, Samantha L. Tornello, Ryan J. Watson

**Affiliations:** ^1^ Department of Human Development and Family Studies The Pennsylvania State University University Park Pennsylvania USA; ^2^ Department of Family Science University of Maryland College Park Maryland USA; ^3^ Edna Bennett Pierce Prevention Research Center The Pennsylvania State University University Park Pennsylvania USA; ^4^ Ross and Carol Nese College of Nursing The Pennsylvania State University University Park Pennsylvania USA; ^5^ Department of Psychology The University of Hong Kong Hong Kong China; ^6^ Department of Human Development and Family Sciences University of Connecticut Storrs Connecticut USA

**Keywords:** intersectional microaggressions, latent profile analysis, mental health, sexual and gender minority youth of color, SOGI‐specific parental responses

## Abstract

Sexual and gender minority youth of color (SGM YOC) face compounded mental health disparities due to oppression experienced at the intersection of racism, cissexism, and heterosexism. Parental responses to youth's sexual orientation and gender identity (SOGI) are critical in shaping youth mental health outcomes, yet prior research has often simplified these responses into a unidimensional acceptance–rejection continuum. Using data from the 2022 LGBTQ National Teen Survey (*n* = 3187 SGM YOC), this study challenged such an oversimplified conceptualization by exploring heterogeneous patterns of parental responses to children's SOGI among SGM YOC. Further, we also examined the distinct implications of each identified profile for SGM YOC's mental health problems and how the association between intersectional microaggressions and mental health problems might vary across the identified profiles. Three distinct SOGI‐specific parental response profiles were found: *High Rejection Low Acceptance Response* (24.10%), *Low Rejection Low Acceptance Response* (50.30%), and *Low Rejection High Acceptance Response* (25.60%). Profile membership varied by race/ethnicity, sex assigned at birth, gender identity, sexual orientation, and caregiver education level. Youth in the *High Rejection Low Acceptance Response* profile reported significantly higher levels of mental health problems compared with those in the *Low Rejection Low Acceptance Response* and *Low Rejection High Acceptance Response* profiles. SOGI‐specific parental response profile membership moderated the association between intersectional microaggressions and mental health problems. Intersectional microaggressions were more strongly associated with mental health problems among youth in the *Low Rejection Low Acceptance Response* profile than among those in the *High Rejection Low Acceptance Response* profile, with higher overall mental health problems in the latter profile. By identifying diverse patterns of SOGI‐specific parental responses and the differential consequences for understanding how SGM YOC's intersectional minority stress relates to mental well‐being, our findings underscore the importance of culturally responsive interventions that engage parental figures as active agents of affirmation, support, and socialization, ultimately fostering SGM YOC's positive development amid intersecting systems of oppression.

## INTRODUCTION

Sexual and gender minority (SGM) youth consistently face elevated risks for adverse mental health outcomes compared with their cisgender heterosexual peers, including depression, anxiety, and posttraumatic stress disorder (National Academies of Sciences, Engineering, and Medicine, 2020), with substantial within‐group heterogeneity shaped by intersectional social positions (Bostwick et al., 2014; Fox et al., 2020; Watson et al., 2024). These mental health inequities may be further exacerbated by recent waves of discriminatory legislation in the United States that explicitly target SGM and racially/ethnically minoritized youth (Levengood & Hadland, 2022; Mallory, 2025). At the same time, research has documented a declining age of identity disclosure among SGM youth compared with previous generations (Russell & Fish, 2019), which may increase their exposure to peer victimization and family rejection during a developmentally sensitive period for the onset of mental health disorders and substance use behaviors (Bishop et al., 2025; Chan & Suen, 2023). This shift toward earlier identity development, coupled with an increasingly hostile sociopolitical climate, highlights the critical role of parents in safeguarding SGM youth from institutional stigma and supporting their well‐being and positive development.

### SOGI‐specific parental acceptance and rejection among SGM YOC

Parental responses to children's sexual orientation and gender identity (SOGI) are critical factors influencing health and well‐being among SGM youth. SOGI‐specific parental rejection is associated with greater mental health problems, suicidality, and substance abuse (Gamarel et al., 2020; Ryan et al., 2009), whereas parental acceptance predicts lower risk of psychological and behavioral difficulties (Newcomb et al., 2012; Olson et al., 2016; Ryan et al., 2010), as well as flourishing (Weeks et al., 2025). Historically, scholarship on parental responses to SOGI has largely been situated along an acceptance–rejection unidimensional continuum, wherein parental responses are classified as either accepting or rejecting of youth SGM identity rather than distinct or co‐occurring constructs (Silva & Orellana‐Calderón, 2025). However, emerging research has challenged this dichotomous framework, which may oversimplify the complexity of SOGI‐specific parental responses (Allen et al., 2022; Hanna‐Walker et al., 2024; Reczek, 2016). For example, beyond outright rejection or unconditional support, some parents may convey mixed messages regarding their children's SOGI (Allen et al., 2022; Reczek, 2016), maintain ambiguity without providing affirmation or clarification (McGuire et al., 2016), or grieve the loss of a child fulfilling certain cisheteronormative expectations (Toomey et al., 2025). Drawing on emotionally focused family therapy work with parents of sexual minority youth, Liow and Huang (2026) proposed a triadic framework of parental responses based on parental intentions and their alignment with children's attachment needs, delineating attuned and affirming responses, overinvolved or controlling actions, and emotionally distant responses.

Quantitative work has begun to reflect heterogeneous patterns of SOGI‐specific parental responses. For example, Clark et al. (2022) identified three distinct patterns of parental reactions to SGM children, with the majority of parents (74.1%) exhibiting a positive response characterized by low levels of rejection indicators and high levels of acceptance indicators, followed by mixed (10.7%) and predominantly negative (15.1%) reaction patterns. However, this emerging literature has relied largely on samples of White SGM youth. Similar configurations and prevalence of parental responses may not generalize to SGM youth of color (YOC), whose family disclosure experiences may be shaped by distinct cultural frameworks and family norms (Koken et al., 2009; Marx et al., 2024; Rosario et al., 2004). For instance, qualitative work has documented that gender norms embedded in Latine cultural values (e.g., *marianismo*, *machismo*; Adames & Chavez‐Dueñas, 2017) may pose challenges for parental acceptance of sexual minority children, whereas other Latine familial values (e.g., *familismo*; Arredondo et al., 2014) and spiritual beliefs may help Latine parents to reconcile internal conflicts and facilitate acceptance over time (Abreu, Gonzalez, et al., 2020). Asian sexual minority youth reported that their parents cautiously avoided discussions of sexuality both before and after identity disclosure, reflecting broader cultural taboos surrounding sexual minority identity in many Asian contexts (Rana et al., 2024). As culture‐specific factors and prior racial–ethnic socialization processes may shape parent–child dynamics surrounding SOGI‐related topics, it is essential to quantitatively identify distinct typologies of SOGI‐specific parental responses within SGM YOC populations. Such work can yield unique insights into the family environments in which SGM YOC are culturally embedded and help identify subgroups of SGM YOC who may benefit from targeted support.

### Demographic predictors of SOGI‐specific parental response profiles

SOGI‐specific parental responses in the general population vary across youth with distinct social identities. In the present study, we focus on variation by age, race/ethnicity, sex assigned at birth, gender identity, sexual orientation, and caregiver education level among SGM YOC given existing empirical evidence. Specifically, prior research indicates that older youth tend to report lower levels of parental support and greater parental difficulty accepting their SOGI (Huebner et al., 2019; McCurdy et al., 2023). In terms of race and ethnicity, although much of the literature has relied on White youth as a reference group in comparison with SGM YOC, emerging evidence highlights meaningful heterogeneity in how cultural values within racially and ethnically diverse communities may shape SOGI‐specific parental responses. For instance, one study found that Latine sexual minority youth felt more comfortable with family members knowing their SOGI than their Black peers (Rosario et al., 2004), which authors attributed to Latine cultural values of *familismo*. *Familismo* is a central Latine cultural value that emphasizes familial support, obligations to maintain cohesion and solidarity, and the prioritization of family harmony over individual needs (Arredondo et al., 2014). These values may influence SOGI‐specific parental responses by fostering ongoing family connection and efforts to preserve relational bonds with Latine youth (Abreu, Gonzalez, et al., 2020; Abreu, Riggle, & Rostosky, 2020). In many Asian cultural contexts, the hegemony of cisheterosexuality may be historically reinforced by Confucian values of filial piety, which emphasize patrilineal continuity through reproduction in heterosexual marriage (Li & Patterson, 2022). In the context of such deeply rooted cultural norms, qualitative work has described a tacit silence that often emerges between Asian and Asian American parents and their children after disclosure (Ulep, 2011; Wei, 2023).

Youth's sex and gender are also relevant to how parents react to their children's identity disclosure. Male young people often report more negative parental reactions than female youth (McCurdy et al., 2023; Ryan et al., 2009). Overall, research suggests that cisgender sexual minority youth are more likely to receive affirming parental responses compared with transgender and nonbinary youth (Gamarel et al., 2020; Hanna‐Walker et al., 2024; McCurdy et al., 2023). Findings regarding sexual orientation differences in SOGI‐specific parental responses are somewhat inconsistent. Some studies indicate that lesbian or gay youth experience higher levels of parental rejection than bisexual or queer youth (Clark et al., 2022), whereas other research suggests that youth with plurisexual identities (e.g., bisexual, pansexual) report less family support for their sexual orientations than their monosexual counterparts (i.e., lesbian or gay; Abreu et al., 2024; Gamarel et al., 2020; Nath et al., 2025). In addition to youth's own identity characteristics, parents' educational attainment has been linked to more positive responses to children's SOGI (Clark et al., 2022; Hanna‐Walker et al., 2024). Collectively, these findings are derived largely from predominantly White SGM samples and may not readily apply to SGM YOC, underscoring the need for a comprehensive examination of demographic predictors of SOGI‐specific parental response profiles within this population.

### Mental health problems across SOGI‐specific parental response profiles

SOGI‐specific parental responses have typically been conceptualized as either rejecting or accepting, with each demonstrating well‐established implications for the mental health of SGM YOC (Nath et al., 2025; Ryan et al., 2009). However, this dichotomous framework leaves unanswered questions regarding the mental health implications of parental responses that fall between or extend beyond these categories, such as responses characterized by the coexistence of acceptance and rejection or by the absence of both. Addressing this gap, Allen et al. (2022) employed latent profile analysis to identify distinct patterns of transgender adults' gender‐related experiences with their family‐of‐origin and their associations with mental health. Their analysis yielded five family environment profiles: *disengaged*, *embracing and affirming*, *repudiating*, *moderate family ambiguity*, and *high family ambiguity*. Using this nuanced typology, the authors found that individuals from *embracing and affirming* families exhibited the most favorable mental health outcomes, whereas the two *family ambiguity* profiles, marked by the presence of supportive and dismissive behaviors toward gender identity, were associated with mental health outcomes comparable to those observed among individuals from *repudiating* families. Similarly, Clark et al. (2022) examined patterns of SOGI‐specific parental responses in relation to children's mental health outcomes. Their findings indicated that negative responses were associated with substantially higher odds of children's anxiety and depression compared with positive responses.

Although these studies have investigated rejection and acceptance as distinct or co‐occurring family processes, their findings are based on parental responses to predominantly White SGM adult children. As such, patterns of SOGI‐specific parental responses and their mental health correlates among SGM persons of color during adolescence remain unknown. This line of inquiry is particularly warranted given that SGM YOC face heightened risks for mental health challenges (Bostwick et al., 2014; Fox et al., 2020; Watson et al., 2024), and adolescence is a critical developmental period for both the onset and development of mental disorders, as well as the promotion of mental well‐being (Kessler et al., 2005). Developmentally informed and culturally responsive interventions that target specific, modifiable parental behaviors and attitudes—beyond acceptance and rejection alone—can have important implications for supporting the mental health of SGM YOC.

### SOGI‐specific parental response profile membership as a potential moderator of the association between intersectional microaggressions and mental health problems

Minority stress theory attributes health disparities between SGM individuals and their cisgender, heterosexual peers to oppression‐based stressors (e.g., prejudice and stigma) directed toward socially stigmatized SOGI status, above and beyond general life stressors (Brooks, 1981; Meyer, 2003). Intersectionality theory challenges the idea that oppression‐based stressors arise from singular marginalized identities and instead emphasizes how multiple, intersecting social positions jointly shape lived experiences and well‐being (Crenshaw, 1989). For instance, many SGM people of color (POC) report experiences of invalidation, disempowerment, and exclusion within LGBTQ spaces that often privilege White SGM individuals (Noyola et al., 2020; Parmenter et al., 2021), encounter exoticized and objectifying stereotypes in White‐dominated social contexts (Weber et al., 2018), and feel compelled to distance themselves from their racial and ethnic communities to avoid homonegativity (Ghabrial, 2017). Accordingly, SGM YOC's experiences of oppression at the intersection of racism, cissexism, and heterosexism have been linked to poorer mental health (Abreu et al., 2023; Jackson et al., 2020). Adopting this intersectional lens, a growing body of research has attended to covert and subtle behaviors or statements that convey hostile or derogatory messages based on the intersections of race and ethnicity, gender identity, and sexual orientation among SGM POC, commonly referred to as *intersectional microaggressions* (Balsam et al., 2011; Fattoracci et al., 2021). These intersectional microaggressions are prevalent in the daily lives of SGM POC across multiple contexts and have been shown to exert significant negative effects on mental health (see Fattoracci et al., 2021 for a review).

In addition to their direct effects on mental health outcomes, SOGI‐specific parental responses are theorized to function as a critical coping resource that shapes how oppression‐based stressors influence youth mental health (Goldbach & Gibbs, 2017; Meyer, 2003). Empirical evidence indicates that SOGI‐specific support from family and friends buffers the associations between identity‐based discrimination and victimization and mental health among SGM youth (Doty et al., 2010; Hershberger & D'Augelli, 1995), whereas parent–child conflict exacerbates these associations (Freitas et al., 2016). More recently, two studies specifically examined the moderating role of SOGI‐specific family acceptance in the association between *intersectional microaggressions* and mental health, documenting a protective buffering effect among Latine SGM youth (Abreu et al., 2023) and queer Asian American young adults (Chong et al., 2025). However, these studies exclusively operationalized SOGI‐specific family responses in binary terms (e.g., acceptance versus rejection; positive versus negative). Distinct configurations of parental acceptance and rejection may differentially shape how intersectional microaggressions are associated with youth mental health. Investigating the moderating role of SOGI‐specific parental response profile membership on the association between intersectional microaggressions and mental health can inform culturally responsive interventions by highlighting specific parental behaviors and attitudes that best support the positive development of SGM YOC. Furthermore, this line of inquiry attends to emerging efforts to reconceptualize positive youth development through a healing‐centered, intersectionality‐informed framework that foregrounds the structural conditions that shape the lives of marginalized youth while simultaneously emphasizing the strengths, resources, and relational contexts that support healing and thriving amid persistent and intersecting forms of oppression (Thomas et al., 2025). The present study contributes to this paradigm by examining mental health outcomes in relation to intersectional microaggressions and identifying how this relationship varies across distinct conditions of SOGI‐specific parental responses. In doing so, we extend understandings of how family‐based assets embedded within culturally situated relational contexts may foster resilience and promote healing among multiply marginalized youth.

### The current study

The present study sought to extend the traditional acceptance–rejection dichotomy prevalent in scholarship on predominantly White family dynamics surrounding SOGI by documenting distinct types of SOGI‐specific parental responses among SGM YOC, treating acceptance and rejection as independent constructs. To capture the complex social positions occupied by SGM YOC across different family environments and to elucidate their mental health implications, we examined demographic predictors of profile membership as well as mental health outcomes associated with the identified profiles. Finally, guided by minority stress theory and intersectionality theory, we investigated the moderating role of SOGI‐specific parental response profiles in the association between intersectional microaggressions and mental health. Such an investigation could reveal whether heterogeneous parental response patterns may protect some subgroups of SGM YOC, while exacerbating risk for others, when exposed to intersectional microaggressions. In sum, the current study utilized a person‐centered approach to address four exploratory research questions:

Are there meaningfully distinct profiles of SOGI‐specific parental acceptance and rejection among SGM YOC?

Which demographic characteristics (i.e., age, race/ethnicity, sex assigned at birth, gender identity, sexual orientation, and caregiver education level) predict profile membership?

How do SGM YOC's mental health differ by profile membership?

Does the association between intersectional microaggressions and mental health vary by profile membership among SGM YOC?

## METHOD

### Participants and procedures

We drew data from the *2022 LGBTQ National Teen Survey* collected between February and October 2022. Youth were eligible to participate if they identified as sexual and/or gender minority, were between 13 and 18 years of age, and resided in the United States at the time of survey completion. Participants were recruited primarily through social media advertisements, with additional in‐person and online outreach conducted in partnership with the Human Rights Campaign (HRC). Multiple verification strategies were implemented to ensure data integrity and prevent fraudulent or automated responses (Watson et al., 2024). Youth who were verified and completed the full survey received a $5 Amazon or Starbucks gift card. Informed electronic assent was obtained from all participants under 18, electronic consent from youth aged 18, and parental consent was waived. Survey measures were developed in collaboration with HRC and its youth advisory members to ensure that they accurately reflected youths' lived experiences and were understandable to participants. Study procedures were approved by the Institutional Review Board at the University of Connecticut.

A total of 17,578 SGM youth met the inclusion criteria and completed the original survey. For the current study, we restricted the sample to SGM YOC who had disclosed their sexual orientation and/or gender identity to at least one caregiver. Accordingly, participants who identified as White and non‐Latine (*n* = 11,459), cisgender heterosexual (*n* = 47), or who had not disclosed their SGM identity to a caregiver (*n* = 911) were excluded. We excluded non‐disclosed youth because prior research has identified mean‐level differences in parental acceptance and rejection indicators between disclosed and non‐disclosed SGM youth (Pollitt et al., 2023). To focus on meaningful parental response patterns, we further excluded participants who were missing on all six parental response indicators (*n* = 1974). These criteria resulted in a final analytic sample of 3187 SGM YOC. Participants in the analytic sample were, on average, 15.85 (*SD* = 1.48) years old. A majority of participants identified as plurisexual (53.59%), transgender and nonbinary (55.60%), and were assigned female at birth (64.04%). Regarding racial and ethnic identity, many participants identified as Latine racial minority (27.80%), Biracial or Multiracial (23.57%), or Latine White (21.37%). Additional demographic information is presented in Table 1.

**TABLE 1:** Demographic characteristics of sexual and gender minority youth of color (*n* = 3187).

Variables	*M* (*SD*) or *n* (%)
Age	15.85 (1.48)
Sex assigned at birth	
Female	2021 (64.04%)
Male	1119 (35.46%)
Intersex	16 (0.51%)
Race/Ethnicity	
Latine Racial Minority	882 (27.80%)
Latine White	678 (21.37%)
American Indian/Alaska Native/Pacific Islander	69 (2.17%)
Asian or Asian American	307 (9.68%)
Black or African American	416 (13.11%)
Biracial/Multiracial	748 (23.57%)
Other Racial/Ethnic Minority	73 (2.30%)
Gender Identity	
Cisgender girl	489 (15.39%)
Cisgender boy	634 (19.95%)
Transgender girl	188 (5.92%)
Transgender boy	615 (19.35%)
Nonbinary	969 (30.40%)
Other gender identities^a^	283 (8.88%)
Sexual orientation	
Lesbian/Gay	987 (30.97%)
Bisexual	917 (28.77%)
Pansexual	472 (14.81%)
Queer	319 (10.01%)
Asexual	210 (6.59%)
Questioning	88 (2.76%)
Heterosexual	44 (1.38%)
Other sexual orientations^b^	150 (4.71%)

*Note*: Not all numbers will total to 100% due to rounding. Participants' sex assigned at birth, gender identity, and race/ethnicity have missing values, so a valid percentage was used for these characteristics.

^a^ Other gender identities include but are not limited to questioning, agender, xenogender, transfeminine, and transmasculine.

^b^ Other sexual orientations include but are not limited to heterosexual, questioning, abrosexual, demisexual, and omnisexual. Due to the categorization logic applied to the race/ethnicity variable, participants in the following groups are non‐Latine: American Indian or Alaska Native, Pacific Islander, Asian or Asian American, Black or African American, Biracial/Multiracial, or Other racial/ethnic minority.

### Measures

#### Demographics

##### Age

Participants reported their age in years in response to the question, “How old are you?”

##### Sex assigned at birth

We assessed participants' sex assigned at birth with the question, “What sex were you assigned at birth?” with response options, “Male,” “Female,” and “Intersex.”

##### Race and ethnicity

We assessed race and ethnicity using two separate items: ethnicity was measured with the question, “Are you Hispanic or Latina/e/o/x?” and race was measured with the question, “What is your race? (select all that apply).” Response options for race included “American Indian or Alaskan Native,” “Asian,” “Black or African American,” “Hawaiian Native/Pacific Islander,” “White,” and “None of these.” For the analytic purpose of the present study, we combined race and ethnicity into a single categorical variable. Participants who identified as Latine were classified as either Latine White if they selected only White for race or Latine racial minority if they selected any racial category other than exclusively White. Participants who did not identify as Latine were categorized as American Indian/Alaska Native/Pacific Islander (combining “Native Hawaiian/Pacific Islander” and “American Indian or Alaska Native”), Asian or Asian American, Black or African American, Other racial/ethnic minority, or Biracial/Multiracial (for those selecting more than one racial category).

##### Gender identity

To assess gender identity, we asked participants to select all identities that applied to them from the following options: “Cisgender boy,” “Cisgender girl,” “Transgender girl,” “Transgender boy,” “Gender nonconforming,” “Genderqueer,” “Gender fluid,” “Nonbinary,” “Questioning,” and “Different identity not listed.” These response options reflect best practices for measuring gender identity among SGM youth and have been used in large epidemiologic surveys with youth samples (DeChants et al., 2021; Rider et al., 2025). Participants who selected “Different identity not listed” were invited to provide a written response, which was subsequently recoded into an existing category when matching or classified as an emerging gender identity. Participants who selected multiple options were prompted to indicate which identity best described them, and we assigned their gender identity with the single option they chose. Based on these responses, gender identity was categorized into six groups: cisgender girl, cisgender boy, transgender girl, transgender boy, nonbinary (combining “Gender nonconforming,” “Genderqueer,” “Gender fluid,” and “Nonbinary”), and other gender identities (combining youth who identified as “Questioning”, youth who selected a “Different identity not listed” that was not captured by existing categories, or youth who selected multiple identities but did not complete the follow‐up item).

##### Sexual orientation

Sexual orientation was measured by the question, “Which of the following BEST describes you? Check one.” Response options included “Gay or lesbian,” “Bisexual,” “Straight/heterosexual,” “Queer,” “Pansexual,” “Asexual,” “Questioning,” and “Something not listed.” Participants who selected “Something not listed” were asked to provide a written response. Written‐in responses that matched an existing category were back coded into an existing category accordingly; all other responses were classified as other sexual orientations.

##### Caregiver education level

Participants reported their caregiver education level by answering the question, “Across all of your caregivers, please indicate the highest level of education that any of your caregivers completed.” Response options ranged from 1 (*Less than high school or GED*) to 6 (*Postgraduate degree or higher*).

#### SOGI‐specific parental acceptance

We assessed SOGI‐specific parental acceptance using three items from the *Family Acceptance Scale* (Pollitt et al., 2023), adapted from the original scale developed by Ryan et al. (2010). These items captured parents' or caregivers' supportive behaviors toward youth SOGI. Participants responded to the prompt, “How often do your parents or caregivers…” followed by three accepting statements (i.e., “say that they like you as you are in regards to being an LGBTQ person,” “say they were proud of you for being an LGBTQ person,” “speak positively about your LGBTQ identity”). Items were rated on a four‐point Likert scale ranging from 1 (*Never*) to 4 (*Often*), with higher scores indicating greater SOGI‐specific parental acceptance. Internal consistency in the current sample was good (Cronbach's α = .86).

#### SOGI‐specific parental rejection

We measured SOGI‐specific parental rejection with three items adapted from the *Family Rejection Scale* (Pollitt et al., 2023; Ryan et al., 2009) that assessed rejecting parental behaviors attributed to youth SOGI. Participants were provided with the same prompt and response options as those used for SOGI‐specific parental acceptance. Rejection items included the following statements: “taunt or mock you because you are an LGBTQ person,” “say negative comments about you being an LGBTQ person,” and “make you feel like you are bad because you are an LGBTQ person.” Higher scores reflected more SOGI‐specific parental rejection. Internal consistency was good in the current sample (Cronbach's α = .86).

#### Intersectional microaggressions

Participants reported their experiences of intersectional microaggressions on the *LGBTQ People of Color Microaggressions Scale* (Balsam et al., 2011). The original 18‐item scale comprises three subscales: *Racism in LGBTQ Communities* (e.g., “Feeling misunderstood by White LGBTQ people”), *Heterosexism in Racial/Ethnic Minority Communities* (e.g., “Not having any LGBTQ people of color as positive role models”), and *Racism in Dating and Close Relationships* (e.g., “Being seen as a sex object by other LGBTQ people because of your race/ethnicity”). Consistent with prior research indicating that the *Racism in Dating and Close Relationships* subscale may not be developmentally relevant for all youth (Mereish et al., 2023, 2025), we only used the first two subscales in the present study (12 items). Participants indicated whether each event had occurred, and if so, the extent to which it bothered them on a five‐point Likert scale ranging from 1 (*Not at all*) to 5 (*Extremely*). Because distress scores were highly right‐skewed, we followed prior work (Mereish et al., 2025) and operationalized intersectional microaggressions using an occurrence score representing the total number of microaggressions endorsed. Internal consistency in the current sample was good (Cronbach's α = .87).

#### Mental health problems

Participants reported their mental health problems on the *Patient Health Questionnaire–4* (PHQ‐4; Kroenke et al., 2009), a brief measure of depressive and anxiety symptoms. Participants reported how often they had been bothered by symptoms of depression and anxiety (e.g., “Feeling nervous, anxious, or on edge,” “Little interest or pleasure in doing things”) during the past 2 weeks. Response options ranged from 0 (*Not at all*) to 3 (*Nearly every day*). We computed a summed score, with higher scores indicating greater mental health problems. Internal consistency in the current sample was good (Cronbach's α = .84).

### Analytic strategies

To address **RQ1**, we conducted latent profile analysis (LPA; Collins & Lanza, 2010) to identify and describe latent profiles of parental acceptance and rejection toward SOGI among SGM YOC. The optimal profile solution was determined by evaluating multiple criteria, including log likelihood (LL), Akaike information criterion (AIC; Akaike, 1974), Bayesian information criterion (BIC; Schwarz, 1978), sample‐size‐adjusted BIC (SABIC; Sclove, 1987), entropy, Vuong‐Lo‐Mendell–Rubin likelihood ratio test (VLMRT; Lo et al., 2001), bootstrapped likelihood ratio test (BLRT; Nylund et al., 2007), profile separation, and theoretical interpretability. Lower values of AIC, BIC, and SABIC indicated better global model fit, whereas higher entropy values (ranging from 0 to 1) reflected greater classification precision (Collins & Lanza, 2010). Significant VLMRT and BLRT results indicated that a model with *k* profiles provided a significant improvement in fit relative to a model with *k − 1* profiles (Nylund et al., 2007). Profile separation was quantified using adapted Cohen's *d*, which reflects the degree of overlap between profile‐specific indicator distributions (Masyn, 2013), as well as holistic comparisons based on profile plots. We handled missing data using full‐information maximum likelihood (FIML) estimation in LPA.

After identifying the optimal profile solution, we examined whether the odds of membership in parental response profiles varied by key demographic factors (**RQ2**). Specifically, age, race/ethnicity, sex assigned at birth, gender identity, sexual orientation, and caregiver education were examined as predictors of profile membership using multinomial logistic regression. We tested these associations using a three‐step approach (i.e., the R3STEP auxiliary command in Mplus), which estimates covariate effects on latent profile membership while accounting for classification uncertainty (Asparouhov & Muthén, 2014). To assess the robustness of our findings given the relatively small sample sizes of “questioning” and “heterosexual” youth, we conducted sensitivity analyses in which these groups were combined with the “other sexual orientations” category. The same multinomial logistic regression models were estimated using this alternative categorization.

To address **RQ3**, we used the three‐step Bolck, Croon, and Hagenaars (BCH) method to examine associations between latent profile membership and SGM YOC's mental health problems (Bolck et al., 2004). This multistep approach assigns individuals to their most likely latent profile based on posterior probabilities and treats profile membership as a known categorical variable in further analyses while incorporating classification uncertainty. To examine differences in mental health across parental response profiles, we estimated profile‐specific means of mental health problems, controlling for demographic covariates included in RQ2. Results are presented as pairwise comparisons of estimated means across profiles with Bonferroni correction. Effect sizes are reported using Cohen's *d*, with values of approximately 0.20, 0.50, and 0.80 interpreted as small, medium, and large, respectively (Cohen, 1988). To address **RQ4**, we then extended the model specified in RQ3 to examine whether latent profile membership moderated the association between intersectional microaggressions and mental health problems by including an interaction between profile membership and intersectional microaggressions, consistent with recommended procedures for testing moderation with latent profile membership (Bray et al., 2023). We conducted data management in R version 4.2.2 (R Core Team, 2022) and all analyses in Mplus version 8.3 (Muthén & Muthén, 1998–2019).

## RESULTS

### RQ1: Latent profiles of SOGI‐specific parental acceptance and rejection

We estimated latent profile models with one to six profiles to determine the optimal number of profiles. Model fit indices for each solution are presented in Table 2. Entropy values indicate high classification quality for all models (range: 0.91–0.95). Although the LL, AIC, BIC, and SABIC continued to decrease with each successive model, the reductions became noticeably smaller with the addition of profiles beyond four. The *p*‐value of BLRT and VLMRT remained significant for the two‐ through five‐profile model, but became nonsignificant for the six‐profile model. Although the five‐profile solution demonstrated improved statistical fit, one profile comprised only 5.30% of the sample and was not qualitatively distinct from existing profiles, raising concerns about stability and interpretability. Therefore, we did not retain the five‐profile solution for further consideration.

**TABLE 2:** Model fit indices for latent profile models with one to six profiles (*n* = 3187).

K	Log likelihood	AIC	BIC	SABIC	Entropy	VLMRT	BLRT	Sample size percentage per profile
1	−28311.46	56646.93	56719.73	56681.60				
2	−25078.93	50195.86	50311.13	50250.75	0.95	<.001	<.001	72.58%; 27.42%
**3**	**−22991.57**	**46035.14**	**46192.87**	**46110.26**	**0.93**	**<.001**	**<.001**	**50.30%; 25.60%; 24.10%**
4	−22286.53	44639.06	44839.26	44734.41	0.95	<.001	<.001	42.93%; 23.16%; 22.80%; 11.11%
5	−21680.16	43440.33	43683.00	43555.90	0.93	.003	.004	37.18%; 23.56%; 19.89%; 14.06%; 5.30%
6	−21270.93	42635.85	42920.99	42771.65	0.91	.067	.069	30.34%; 19.83%; 15.88%; 14.53%; 13.81%; 5.62%

*Note*: Bold values indicate the selected latent profile model.

Abbreviations: AIC, Akaike information criterion; BIC, Bayesian information criterion; BLRT, Bootstrapped Likelihood Ratio Test; SABIC, sample‐size‐adjusted Bayesian information criterion; VLMRT, Vuong‐Lo–Mendell–Rubin likelihood ratio test.

Thus, we closely examined both three‐ and four‐profile solutions to inform the model enumeration process and theoretical interpretation. The four‐profile model largely replicated the structure of the three‐profile model, with the fourth profile characterized by low levels of parental rejection and moderate levels of parental acceptance, which appeared to represent an “average” group relative to two profiles in the three‐profile model (i.e., Profiles 2 and Profile 3, described below). To evaluate the distinctiveness of the additional profile in the four‐profile model (i.e., profile separation), we compared the indicator‐level mean differences between the fourth profile and each of the other profiles. Five out of the six indicators failed to reach a satisfactory adjusted Cohen's *d* of 2 when comparing the fourth profile to the other two profiles that closely resembled Profile 2 and Profile 3 in the three‐profile model, indicating insufficient profile separation (Masyn, 2013). Given the lack of clear separation and interpretability in the four‐profile solution, along with the limited added value of more complex solutions, the three‐profile model was selected as the most parsimonious and theoretically meaningful representation for subsequent analyses.

**FIGURE 1:**
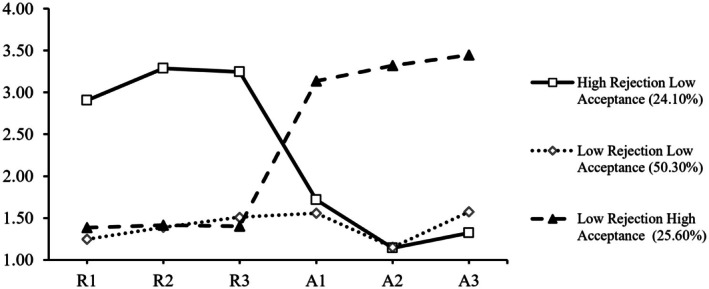
Latent profiles of SOGI‐specific parental acceptance and rejection (*n* = 3187). R1 = rejection item 1: Taunt or mock you because you are an LGBTQ person; R2 = rejection item 2: Say negative comments about you being an LGBTQ person; R3 = rejection item 3: Make you feel like you are bad because you are an LGBTQ person; A1 = acceptance item 1: Say that they like you as you are in regards to being an LGBTQ person; A2 = acceptance item 2: Say they were proud of you for being an LGBTQ person; A3 = acceptance item 3: Speak positively about your LGBTQ identity. Means of profile indicators range from 1 to 4.

Profile prevalences and indicator means conditional on the three profiles are presented in Figure 1. Ultimately, three profiles were labeled respectively as follows. Profile 1: *High Rejection Low Acceptance Response* profile (24.10% of participants), characterized by lower mean values of SOGI‐specific parental acceptance indicators and higher mean values of parental rejection indicators; Profile 2: *Low Rejection Low Acceptance Response* profile (50.30% of participants), characterized by lower mean values of all SOGI‐specific parental acceptance and rejection indicators; Profile 3: *Low Rejection High Acceptance Response* profile (25.60% of participants), characterized by higher mean values of SOGI‐specific parental acceptance indicators and lower mean values of parental rejection indicators.

### RQ2: Demographic predictors of profile membership

Multinomial logistic regressions with the *Low Rejection Low Acceptance Response* profile as the reference group are presented in Table 3. Results indicated that race/ethnicity, sex assigned at birth, gender identity, sexual orientation, and caregivers' education level predicted profile membership. Compared with Latine racial minority youth, Asian or Asian American (*OR* = 0.36, *p* < .01) and other non‐Latine racial/ethnic minority youth (*OR* = 0.50, *p* = .02) were less likely to be in the *Low Rejection High Acceptance Response* profile relative to the *Low Rejection Low Acceptance Response* profile. Youth assigned male at birth were less likely than those assigned female at birth to be in the *High Rejection Low Acceptance Response* profile (*OR* = 0.39, *p* < .01) and the *Low Rejection High Acceptance Response* profile (*OR* = 0.76, *p* = .02) than the *Low Rejection Low Acceptance Response* profile. Compared with cisgender boys, cisgender girls were less likely to be in the *High Rejection Low Acceptance Response* profile (*OR* = 0.32, *p* < .01) and the *Low Rejection High Acceptance Response* profile (*OR* = 0.66, *p* < .01); transgender boys were more likely to be in the *High Rejection Low Acceptance Response* profile (*OR* = 1.90, *p* < .01) and the *Low Rejection High Acceptance Response* profile (*OR* = 1.66, *p* = .02); transgender girls were more likely to be in the *High Rejection Low Acceptance Response* profile (*OR* = 2.18, *p* = .02) than the *Low Rejection Low Acceptance Response* profile. Compared with lesbian or gay youth, bisexual, asexual, and questioning youth were more likely to be in the *Low Rejection Low Acceptance Response* profile than both *High Rejection Low Acceptance Response* (bisexual: *OR* = 0.78, *p* = .04; asexual: *OR* = 0.66, *p* = .02; questioning: *OR* = 0.39, *p* < .01) and *Low Rejection High Acceptance Response* profile (bisexual: *OR* = 0.67, *p* < .01; asexual: *OR* = 0.48, *p* < .01; questioning: *OR* = 0.52, *p* < .01). Pansexual and queer youth were less likely than lesbian or gay youth to be in the *Low Rejection High Acceptance Response* profile than the *Low Rejection Low Acceptance Response* profile (pansexual: *OR* = 0.72, *p* = .01; queer: *OR* = 0.73, *p* = .04). Youth with caregivers who had higher (versus lower) levels of educational attainment were less likely to be in the *High Rejection Low Acceptance Response* profile than in the *Low Rejection Low Acceptance Response* profile (*OR* = 0.92, *p* = .02).

**TABLE 3:** Demographic predictors of profile membership (*n* = 2879).

	Ref: Low rejection					
	Low acceptance response (profile 2)					
	High rejection low acceptance response (profile 1)			Low rejection high acceptance response (profile 3)		
	OR	*SE*	*p*	OR	*SE*	*p*
Age	1.01	0.04	0.81	0.99	0.03	0.81
Race/Ethnicity (ref: Latine Racial Minority)						
Latine White	1.07	0.17	0.66	1.15	0.16	0.35
American Indian/Alaska Native/Pacific Islander	1.52	0.58	0.37	1.34	0.47	0.47
Asian or Asian American	1.35	0.26	0.18	**0.36**	**0.08**	**<0.01**
Black or African American	1.05	0.19	0.79	0.90	0.14	0.49
Biracial/Multiracial	1.25	0.19	0.19	0.97	0.13	0.81
Other Racial/Ethnic Minority	1.78	0.57	0.17	**0.50**	**0.21**	**0.02**
Sex assigned at birth (ref: Female)						
Male	**0.39**	**0.06**	**<0.01**	**0.76**	**0.10**	**0.02**
Intersex	1.05	0.85	0.95	2.10	1.53	0.47
Gender identity (ref: Cisgender Boy)						
Cisgender girl	**0.32**	**0.06**	**<0.01**	**0.66**	**0.11**	**<0.01**
Transgender boy	**1.90**	**0.30**	**<0.01**	**1.66**	**0.27**	**0.02**
Transgender girl	**2.18**	**0.51**	**0.02**	1.51	0.34	0.13
Nonbinary	0.91	0.15	0.56	1.05	0.16	0.75
Other gender identities	1.63	0.45	0.17	1.40	0.39	0.30
Sexual orientation (ref: Gay/Lesbian)						
Bisexual	**0.78**	**0.11**	**0.04**	**0.67**	**0.08**	**<0.01**
Pansexual	0.91	0.15	0.56	**0.72**	**0.11**	**0.01**
Queer	0.97	0.18	0.85	**0.73**	**0.13**	**0.04**
Asexual	**0.66**	**0.15**	**0.02**	**0.48**	**0.11**	**<0.01**
Questioning	**0.39**	**0.15**	**<0.01**	**0.52**	**0.16**	**<0.01**
Heterosexual	1.13	0.52	0.80	1.48	0.62	0.44
Other sexual orientations	0.83	0.21	0.40	0.77	0.19	0.24
Caregiver education level	**0.92**	**0.03**	**0.02**	1.05	0.03	0.17

*Note*: Significant predictors (estimates *p* < .05) are bolded for emphasis.

Abbreviations: OR, odds ratio; SE, Standard Error; Ref, Reference Group.

In sensitivity analyses examining whether combining “questioning” and “heterosexual” youth with the “other sexual orientations” category yielded a similar pattern of results (see Table S1), we found that the combined “other sexual orientations” group was more likely to be classified in the *Low Rejection Low Acceptance* Response profile relative to the *High Rejection Low Acceptance* profile compared with lesbian or gay youth. Considering this pattern in relation to the main results of RQ2, this finding suggests that the distinct experiences of questioning youth may be driving this effect and that combining these groups may obscure meaningful differences in parental responses to youths' sexual orientation.

### RQ3: SGM YOC's mental health problems by profile membership

After controlling for demographic covariates (i.e., youth age, race/ethnicity, sex assigned at birth, gender identity, sexual orientation, and caregiver education level), SGM YOC with parents in the *Low Rejection Low Acceptance Response* profile (*Mean* = 6.37, *SD* = 3.33, estimated mean difference = 1.58, *p* < .01, Cohen's *d* = 0.48) and the *Low Rejection High Acceptance Response* profile (*Mean* = 6.57, *SD* = 3.29, estimated mean difference = 1.38, *p* < .01, Cohen's *d* = 0.42) reported significantly less mental health problems than those with parents in the *High Rejection Low Acceptance Response* group (*Mean* = 7.95, *SD* = 3.21). No significant differences in mental health problems were observed between youth in the *Low Rejection Low Acceptance Response* and *Low Rejection High Acceptance Response* profiles (estimated mean difference = −0.20, *p* = .23, Cohen's *d* = −0.06).

### RQ4: Differential associations between intersectional microaggressions and mental health problems by profile membership

Before conducting latent profile moderation analyses, we first confirmed that intersectional microaggressions were associated with more mental health problems among SGM YOC (*b* = 0.22, *SE* = 0.02, *p* < .01). Table 4 shows results from the moderated linear regression model using the *Low Rejection Low Acceptance Response* profile as the reference group, such that effects of membership in the *High Rejection Low Acceptance Response* and *Low Rejection High Acceptance Response* profiles were compared against this group.

**TABLE 4:** Associations between intersectional microaggressions and mental health problems by latent profile membership (*n* = 2857).

	β	*SE*	*p*
Intercept (Profile 2)	**4.57**	**0.18**	**<0.01**
Intersectional microaggressions (IM; Profile 2)	**0.22**	**0.03**	**<0.01**
Latent profiles of parental responses			
Profile 2: Low Rejection Low Acceptance	Ref		
Profile 1: High Rejection Low Acceptance	**2.25**	**0.40**	**<0.01**
Profile 3: Low Rejection High Acceptance	0.56	0.32	0.08
Moderation			
IM (Profile 2)	Ref		
IM (Profile 1 vs. Profile 2)	**−0.10**	**0.05**	**0.04**
IM (Profile 3 vs. Profile 2)	−0.06	0.04	0.14

*Note*: Profile 2 (*Low Rejection Low Acceptance*) was treated as the reference group in the moderation analysis. Reported profile effects represent mean differences in mental health outcomes relative to the reference profile. Interaction terms represent differences in the conditional slope of intersectional microaggressions (IM) on mental health outcomes between each profile and Profile 2. Statistically significant estimates are presented in bold.

**FIGURE 2:**
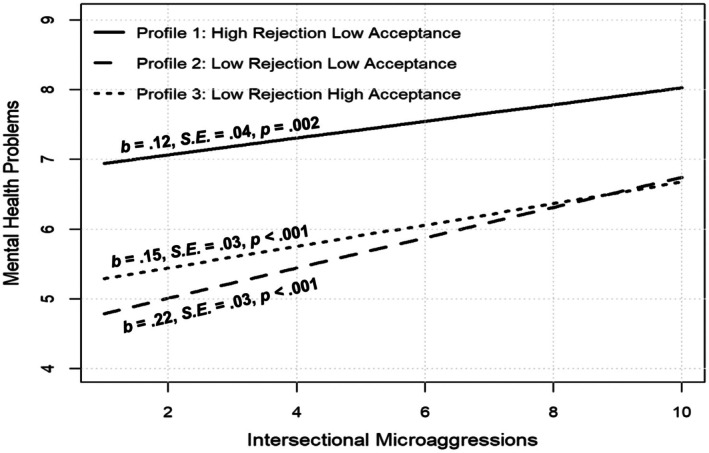
Simple slope illustration for the association between intersectional microaggressions and mental health problems moderated by latent profile membership.

Model results indicated that SOGI‐specific parental response profile membership moderated the association between intersectional microaggressions and mental health problems. Compared with youth in the *High Rejection Low Acceptance Response* profile (*b* = 0.12, *SE* = 0.04, *p* < .01, β = 0.12), the association of microaggressions with mental health problems was more pronounced among youth in the *Low Rejection Low Acceptance Response* profile (∆*b* = −0.10, *SE* = 0.05, *p* = .04). Notably, although the relationship between intersectional microaggressions and mental health problems was weaker among youth in the *High Rejection Low Acceptance Response* profile, their overall levels of mental health problems remained higher than those of youth in other profiles (see Figure 2). The association between intersectional microaggressions and mental health problems did not differ significantly between youth in the *Low Rejection High Acceptance Response* profile (*b* = 0.15, *SE* = 0.03, *p* < .01, β = 0.18) and those in the *Low Rejection Low Acceptance Response* profile (*b* = 0.22, *SE* = 0.03, *p* < .01, β = 0.23; ∆*b* = −0.06, *SE* = 0.04, *p* = .14).

## DISCUSSION

Moving beyond the traditional acceptance–rejection single continuum and conceptualizing parental acceptance and rejection as distinct constructs is pivotal for capturing the complexity of parent–child relationships surrounding SOGI (McGuire et al., 2016; Reczek, 2016). Building on this growing body of scholarship and guided by an intersectional framework attentive to multiple marginalized social identities, the present study drew on a national sample of 3187 SGM YOC to quantitatively derive a typology of SOGI‐specific parental responses based on separate indicators of acceptance and rejection. Following the identification of parental response profiles, we examined demographic correlates of profile membership, mental health outcomes associated with each profile, and whether profile membership moderated the association between intersectional microaggressions and mental health among SGM YOC.

### Distinct profiles of SOGI‐specific parental response

We identified three unique patterns of SOGI‐specific parental responses among SGM YOC. Approximately 24.10% of participants were classified into the *High Rejection Low Acceptance Response* profile (Profile 1), 25.60% into the *Low Rejection High Acceptance Response* profile (Profile 3), and half of the sample (50.30%) into the *Low Rejection Low Acceptance Response* profile (Profile 2). The *High Rejection Low Acceptance Response* profile and *Low Rejection High Acceptance Response* profile largely correspond to the patterns of outright rejection and full acceptance that have been documented in prior research (Ryan et al., 2009, 2010). Notably, although prior research with parents of predominantly White SGM youth has identified parental response patterns similar to those found in our study, the relative prevalence of these profiles differed in the current sample of SGM YOC compared with prior samples of predominantly White SGM youth. In particular, support and affirmation appear to be the most prevalent response pattern among parents of White SGM youth (Clark et al., 2022: 74.1% [Positive Response]; Hanna‐Walker et al., 2024: 41.8% [Positive Parental Response]), whereas rejection is less common (Clark et al., 2022: 15.1% [Negative Response]; Hanna‐Walker et al., 2024: 15.2% [High Negative Parental Response]). The lower prevalence of affirming responses and the relatively higher prevalence of less supportive patterns among parents of SGM YOC may reflect the influence of culturally embedded norms surrounding sexuality and gender expression within racially and ethnically diverse communities in shaping parental responses to SOGI (Cerezo et al., 2020).

In addition, the *Low Rejection Low Acceptance Response* profile identified in our study replicates the *Low Parental Response* profile identified by Hanna‐Walker et al. (2024), characterized by minimal positive and negative SOGI‐specific responses from youth religious parents. It also corresponds to the *Disengaged* profile found by Allen et al. (2022), which reflects low endorsement of both “accepting” and “rejecting” family environment indicators among transgender adults. Together, these findings challenge the acceptance–rejection single continuum of SOGI‐specific parental responses and suggest that silence and emotional distancing around SGM‐related topics—where parents neither provide affirmation nor express overt hostility—may represent a common family experience across various racial and ethnic contexts. The recent wave of anti‐LGBTQ+ executive orders, policy rollbacks, and hostile rhetoric in the United States has deprived many SGM youth of access to affirming healthcare and adversely affected their mental and physical health, with particularly pronounced consequences for SGM YOC and their families (Abbott et al., 2026; Jones, 2024). This rapidly shifting sociopolitical landscape may further contribute to silent parental responses as caregivers attempt to navigate increasingly hostile external environments affecting their children alongside their own internal adjustment processes (Robinson & Brewster, 2016).

The three‐profile model of SOGI‐specific parental responses identified among SGM YOC aligns with a recently proposed triadic caregiving model that advances conceptualizations of parental acceptance and rejection grounded in attachment theory and emotionally focused family therapy (Liow & Huang, 2026). The proposed triadic caregiving model incorporates parents' underlying motivations and their alignment with children's attachment needs into the traditional acceptance–rejection model, thereby reframing SOGI‐specific parental responses into three caregiving types: (1) *matched care*, characterized by attuned and affirming behaviors; (2) *misguided care*, characterized by controlling behaviors driven by parents' beliefs about what is best for the child; and (3) *withdrawn care*, characterized by emotional distancing stemming from personal distress. This framework maps closely onto the *High Rejection Low Acceptance Response* and *Low Rejection High Acceptance Response* profiles identified in the present study, and provides a conceptual lens for understanding the silence and emotional distance captured by the *Low Rejection Low Acceptance* profile. Specifically, parents of SGM YOC are likely to adopt this withdrawn response as self‐protective strategies to navigate intersecting pressures surrounding their children's SOGI, such as anticipated stigma, religious conflict, fear of social judgment, or perceived threats to social standing within their racial and ethnic communities (Cerezo et al., 2020; Liow & Huang, 2026).

### Key demographic predictors of SOGI‐specific parental response profiles

Among the key demographic predictors, race/ethnicity, sex assigned at birth, gender identity, sexual orientation, and caregivers' education were meaningfully associated with profile membership. With respect to race and ethnicity, Asian or Asian American youth were more likely than Latine racial minority youth to report parental responses characterized by *Low Rejection Low Acceptance Response* rather than *Low Rejection High Acceptance Response*. Although silence around sexuality has been documented in both Asian and Latine families (Cerezo et al., 2020; Chan, 1997), certain culturally grounded values may help explain this group difference. In Asian and Asian American contexts, values rooted in Confucian traditions emphasizing moderation, relational harmony, and emotional restraint (e.g., *Zhongyong* [中庸], the Doctrine of the Mean) may contribute to tacit silence between SGM youth and their parents, such that parents may neither affirm nor overtly reject their children's SOGI (Ulep, 2011; Wang et al., 2020; Wei, 2023). In contrast, prior research has documented that Latine cultural values, such as *familismo*, could facilitate more affirming parental responses, probably due to emphases on family unity, emotional connectedness, and unconditional support (Abreu, Gonzalez, et al., 2020). These findings suggest that, while parents in both groups may navigate tensions between cultural norms and children's SOGI, differences in the emphasis on emotional expression and family dynamics may contribute to the greater likelihood of disengaged responses among Asian families compared with more affirming responses among Latine racial minority families. Future research is promising to directly test these cultural explanations.

SGM YOC assigned male at birth were more likely to experience *Low Rejection Low Acceptance Response* from parents than either the *High Rejection Low Acceptance Response* or *Low Rejection High Acceptance Response*, compared with those assigned male at birth. This finding partially contrasts with, while also extending, prior work based on an acceptance–rejection continuum suggesting that SGM youth assigned male at birth report more negative parental reactions (McCurdy et al., 2023; Ryan et al., 2009). Additionally, compared with cisgender boys of color, cisgender girls of color were more likely to report *Low Rejection Low Acceptance* parental responses relative to the other two response profiles, suggesting a more nuanced intersection of gender norms and sexuality in shaping parental reactions. Transgender boys of color were more likely than cisgender boys of color to be classified in both the *High Rejection Low Acceptance* and the *Low Rejection High Acceptance* profiles, whereas transgender girls of color were more likely to be classified in the *High Rejection Low Acceptance* profile. These patterns are consistent with extant evidence that gender identity is often perceived as a less concealable identity than sexual orientation (Le Forestier et al., 2025), which may prompt parents to adopt more explicit responses to a transgender identity than to a lesbian or gay identity. A general pattern that emerged from sexual orientation predictors is that plurisexual YOC (e.g., bisexual, pansexual) were more likely to be classified in the *Low Rejection Low Acceptance Response* profile, relative to lesbian or gay YOC. This may be due to the greater social visibility and normative understanding of monosexual identities compared with plurisexual identities (Abreu et al., 2024; Nelson, 2020). As a result, parents of plurisexual YOC may be in an ongoing, iterative process of appraisal and meaning‐making regarding their children's sexual identity (Chrisler, 2025), which may manifest in their attitudes and behaviors as disengagement characterized by neither explicit rejection nor affirmation. Alternatively, some plurisexual YOC may be in different‐gender relationships and are perceived as conforming to heteronormative expectations (Cipriano et al., 2023), which can further reduce the likelihood of overt parental responses to their SOGI. We also found that SGM YOC whose caregivers had higher levels of education were less likely to be classified in the *High Rejection Low Acceptance Response* profile than in the *Low Rejection Low Acceptance Response* profile. This finding aligns with prior research linking higher parents' education to more supportive responses to children's SOGI (Clark et al., 2022; Hanna‐Walker et al., 2024). Taken together, patterns of demographic predictors observed in the present study indicate both convergence with prior research and important nuances that warrant interpretation within culturally relevant frameworks.

### Mental health problems across SOGI‐specific parental response profiles

With respect to youth mental health, SGM YOC who experienced *High Rejection Low Acceptance Response* from their parents reported poorer mental health compared with youth in the other two profiles, and no significant differences in mental health were observed between youth assigned to the *Low Rejection High Acceptance Response* profile and those in the *Low Rejection Low Acceptance Response* profile. These findings substantiate prior evidence documenting the direct harm of family rejection on SGM youth's positive development and well‐being (Gamarel et al., 2020; Ryan et al., 2009). Although the specific profile structures differed, our results resonate with findings from Allen et al. (2022), which showed that transgender adults from *Disengaged* family environments exhibited better mental health than those from *Repudiating* families. This pattern of results may suggest that emotional distance between SGM YOC and their parents may reduce exposure to emotionally taxing parent–child interactions shaped by cisheteronormative expectations within culturally prescribed norms—such as traditional filial piety in many Asian cultural contexts (Li & Patterson, 2022) or Latine cultural values including *familismo*, *machismo*, and *marianismo* (Abreu, Gonzalez, et al., 2020)—thereby mitigating associated psychological distress.

### The association between intersectional microaggressions and mental health problems varied systematically across the identified parental response profiles

Furthermore, our findings indicated that the association between intersectional microaggressions and mental health outcomes varied across profiles. Specifically, this relation was stronger among SGM YOC whose parents exhibited *Low Rejection Low Acceptance Response* compared with those with *High Rejection Low Acceptance Response* parents. Although youth in the *Low Rejection Low Acceptance Response* profile demonstrated better mental health than youth in the *High Rejection Low Acceptance Response* profile, they appeared more susceptible to the negative mental health effects of intersectional microaggressions. One possible explanation is that parental emotional distance or disengagement may leave SGM YOC less prepared to cope with stressors arising at the intersection of multiple marginalized identities. It is also plausible that, because the family environment constitutes a critical proximal context for youth development, highly rejecting parental responses may function as a rather salient and direct determinant of youth mental health, potentially outweighing the effects of intersectional microaggressions that SGM YOC are more likely to encounter in more distal ecological contexts, such as school settings (Durwood et al., 2021; Veale et al., 2017).

Although the study did not explicitly reveal a buffering effect of a particular SOGI‐specific parental response pattern, it nonetheless attends to the assertions advanced by the healing‐centered, intersectionality‐based positive youth development framework (Thomas et al., 2025). From this perspective, thriving among SGM YOC is not defined solely by the absence of distress, but by the availability of relational and structural resources that support active coping and healing in the context of ongoing oppression. Considered together, our findings regarding mental health differences across parental response profiles and the moderating role of profile membership suggest that parental disengagement or emotional distance may help youth avoid immediate psychological harm, yet simultaneously limit opportunities for youth to actively process, resist, and reconcile with persistent structural barriers to well‐being. In contrast, unequivocal parental affirmation may play a central role not only in promoting mental health, but also in fostering the conditions under which SGM YOC can engage in effective coping and thriving amid intersecting systems of racism, cissexism, and heterosexism.

### Practical implications

Our findings offer important practical implications for the development of culturally responsive, family‐based interventions for SGM YOC. The SOGI‐specific parental response patterns identified in this study extend beyond binary conceptualizations of acceptance and rejection and underscore the need for practitioners and clinicians to recognize the emotional, behavioral, and attitudinal complexity embedded in parents' responses to their children's SOGI. By attending to these nuanced response patterns, professionals can tailor intervention strategies to the specific needs and challenges associated with each parental response profile, rather than relying on one‐size‐fits‐all approaches. For example, psychoeducational interventions grounded in evidence‐based research may be particularly salient for parents in the *High Rejection Low Acceptance Response* profile, who may express disparaging comments or endorse harmful practices such as conversion efforts based on unscientific beliefs (Shpigel et al., 2015). In contrast, parents in the *Low Rejection Low Acceptance Response*—who may be grappling with internal value conflicts often shaped by rigid religious or cultural norms—may benefit from interventions that validate their emotional experiences while engaging them in collaborative, reflective conversations that help them negotiate a dynamic balance between their beliefs and their children's developmental needs (Liow & Huang, 2026).

Beyond profile‐specific strategies, our findings highlight the importance of situating family‐based interventions within the broader, intersecting social positions and lived experiences of SGM YOC and their families. Clinicians and practitioners should attend to the cultural values that may underlie particular parental response patterns across ethnoracial groups. Developing cultural competence involves recognizing that cultural beliefs can both challenge and support parents' processes of accepting their children's SOGI. Accordingly, practitioners should avoid reinforcing deficit‐based stereotypes and instead draw on culturally grounded assets that can foster dialogue and connection within families (Fish & Ezra, 2023). For example, expressive writing interventions have been shown to support Latine parents in processing complex emotions related to their children's SGM identities within their cultural frameworks, while also helping clinicians better understand culturally specific strengths and challenges that shape pathways to acceptance (Abreu, Riggle, & Rostosky, 2020). Extending and evaluating culturally adapted versions of such interventions in other racially and ethnically diverse contexts represents a promising direction for future research.

Policies and interventions aimed at promoting flourishing among SGM youth of color should therefore move beyond a sole focus on desired developmental outcomes and instead situate youth development within the structural conditions of racism, cissexism, and heterosexism (Thomas et al., 2025). From this perspective, engaging parental figures as active agents of affirmation, support, and socialization is essential for fostering SGM YOC's capacity to grow and thrive amid intersecting systems of oppression.

### Limitations and future directions

Despite unique strengths of the current study, including an intersectional framework, a diverse national sample, and a person‐centered methodological approach that advances understanding of SOGI‐specific parental responses, several limitations and directions for future research warrant consideration. First, SOGI‐specific parental responses in the present study were assessed using six items primarily capturing positive and negative verbal communication. However, prior research indicates that SOGI‐specific acceptance and rejection encompass a broader range of behavioral manifestations, including endorsement of anti‐LGBTQ+ beliefs, conversion efforts, explicit care, and parental advocacy (Silva & Orellana‐Calderón, 2025). As such, future studies employing more comprehensive and multidimensional measures of SOGI‐specific parental responses may identify more nuanced profile structures and yield more tailored implications for intervention.

Second, our examination of demographic predictors of parental response profile membership focused on single social positions (e.g., race/ethnicity, gender identity, sexual orientation) rather than their intersections. Although this approach provides an important first step, it does not fully capture how multiple marginalized identities jointly shape family processes. Future research with larger samples of SGM YOC would enable the use of quantitative intersectional methods, such as multilevel analysis of individual heterogeneity and discriminatory accuracy (Evans et al., 2018), to more rigorously examine how intersecting social positions are associated with differential patterns of SOGI‐specific parental responses.

Third, the current study assessed SOGI‐specific parental responses with frequency‐based indicators at a single time point, thereby capturing parental responses as relatively stable, global patterns rather than as dynamic processes. Prior work suggests that parental responses to youth SOGI are often evolving and may change over time following identity disclosure (Chrisler, 2025). Longitudinal designs that repeatedly assess parental responses across key developmental and relational transitions would provide valuable insight into the stability and change of parental response profiles and clarify whether and how parents move between patterns. Such work would be particularly informative for identifying critical windows and leverage points for family‐based interventions aimed at strengthening parent–child relationships around SOGI‐related topics.

## CONCLUSIONS

Drawing on a national sample of 3187 SGM YOC, this study identified three distinct patterns of SOGI‐specific parental responses: *High Rejection Low Acceptance Response* (24.10%), *Low Rejection Low Acceptance Response* (50.30%), and *Low Rejection High Acceptance Response* (25.60%). These parental response profiles varied by race/ethnicity, sex assigned at birth, gender identity, sexual orientation, and caregivers' education attainment. SGM YOC in the *High Rejection Low Acceptance Response* profile reported significantly higher levels of mental health problems compared with those in the *Low Rejection Low Acceptance Response* and *Low Rejection High Acceptance Response* profiles. Moreover, the association between intersectional microaggressions and mental health problems varied by profile membership. Intersectional microaggressions were more strongly associated with mental health problems among youth in the *Low Rejection Low Acceptance Response* profile than among those in the *High Rejection Low Acceptance Response* profile, with higher overall mental health problems in the latter profile. By identifying diverse patterns of SOGI‐specific parental responses, this study moves beyond the traditional acceptance–rejection single continuum and highlights the complexity of parent–child dynamics surrounding SOGI within racially and ethnically diverse family contexts. Together, these findings underscore the importance of culturally responsive interventions that engage parental figures as active agents of affirmation, support, and socialization, ultimately fostering SGM YOC's positive development amid intersecting systems of oppression.

## AUTHOR CONTRIBUTIONS

**Hongjian Cao:** Conceptualization; validation; writing – review and editing; supervision; resources. **Cara L. Exten:** Methodology; validation; writing – review and editing; resources; supervision. **Shixin Fang:** Conceptualization; writing – original draft; methodology; visualization; writing – review and editing; formal analysis. **Ryan J. Watson:** Investigation; funding acquisition; validation; writing – review and editing; project administration; data curation; resources. **Samantha L. Tornello:** Writing – review and editing; validation; supervision; resources. **Meg D. Bishop:** Conceptualization; methodology; validation; writing – review and editing; supervision; resources.

## FUNDING INFORMATION

The work described herein was supported by a grant T32 DA017629, awarded to Fang by the National Institute on Drug Abuse. Bishop gratefully acknowledges support from grants F32AA030194 by the National Institute on Alcohol Abuse and Alcoholism, P2C‐HD041041 awarded to the Maryland Population Research Center by the *Eunice Kennedy Shriver* National Institute of Child Health and Human Development, and #U48 DP006382 awarded to the University of Maryland Prevention Research Center by the Centers for Disease Control. Watson acknowledges support from the National Institute on Drug Abuse, grant K01DA047918, and the National Institute on Minority Health and Health Disparities grant R01MD015722‐S1.

## CONFLICT OF INTEREST STATEMENT

The authors declared no potential conflicts of interest with respect to the research, authorship, and/or publication of this article.

## ETHICS STATEMENT

This study was performed in line with the principles of the Declaration of Helsinki. Approval was granted by the Institutional Review Board at the University of Connecticut (UConn IRB H21‐0087).

## PATIENT CONSENT STATEMENT

A waiver of parental consent was granted by the study IRB due to the sensitive nature of questions related to youth sexual orientation and gender identity.

## Supporting information


Table S1.

## Data Availability

Data sharing is not applicable to this article as no new data were created or analyzed in this study.
