# Supplementary material for: Profiles of parental response, intersectional microaggressions, and mental health problems among sexual and gender minority youth of color
Source: J Res Adolesc. 2026 Jun 22;36(2):e70219. doi: 10.1111/jora.70219 (PMC13285007; doi:10.1111/jora.70219)
Supplement: Supplementary file 1 — Table S1. [file JORA-36-0-s001.docx]

**Table S1**

*Demographic predictors of profile membership (n = 2,879)*

|  | *Ref: Low Rejection*  *Low Acceptance Response (Profile 2)* | | | | | | | |
| --- | --- | --- | --- | --- | --- | --- | --- | --- |
|  | *High Rejection Low Acceptance Response (Profile 1)* | | |  | | *Low Rejection High Acceptance Response (Profile 3)* | | |
|  | *OR* | *S.E.* | *p* |  | | *OR* | *S.E.* | *p* |
| **Age** | 1.01 | 0.04 | 0.79 | | 0.99 | | 0.03 | 0.85 |
| **Race/Ethnicity (ref: *Latine Racial Minority*)** |  |  |  | |  | |  |  |
| *Latine White* | 1.08 | 0.17 | 0.65 | | 1.16 | | 0.16 | 0.32 |
| *American Indian/Alaska Native/Pacific Islander* | 1.52 | 0.58 | 0.37 | | 1.34 | | 0.47 | 0.46 |
| *Asian or Asian American* | 1.33 | 0.26 | 0.20 | | **0.36** | | **0.08** | **< 0.01** |
| *Black or African American* | 1.04 | 0.19 | 0.82 | | 0.90 | | 0.14 | 0.47 |
| *Biracial/Multiracial* | 1.24 | 0.19 | 0.20 | | 0.96 | | 0.13 | 0.76 |
| *Other Racial/Ethnic Minority* | 1.73 | 0.54 | 0.18 | | **0.49** | | **0.20** | **0.01** |
| **Sex assigned at birth (ref: Female)** |  |  |  | |  | |  |  |
| *Male* | **0.39** | **0.06** | **< 0.01** | | **0.77** | | **0.10** | **0.03** |
| *Intersex* | 1.05 | 0.85 | 0.95 | | 2.10 | | 1.53 | 0.47 |
| **Gender identity (ref: Cisgender Boy)** |  |  |  | |  | |  |  |
| *Cisgender girl* | **0.32** | **0.06** | **< 0.01** | | **0.65** | | **0.11** | **< 0.01** |
| *Transgender boy* | **1.91** | **0.30** | **< 0.01** | | **1.69** | | **0.28** | **0.01** |
| *Transgender girl* | **2.26** | **0.52** | **0.02** | | 1.61 | | 0.35 | 0.08 |
| *Nonbinary* | 0.91 | 0.15 | 0.55 | | 1.05 | | 0.16 | 0.73 |
| *Other gender identities ^a^* | 1.66 | 0.46 | 0.15 | | 1.41 | | 0.39 | 0.29 |
| **Sexual orientation (ref: Gay/Lesbian)** |  |  |  | |  | |  |  |
| *Bisexual* | **0.78** | **0.11** | **0.04** | | **0.67** | | **0.08** | **< 0.01** |
| *Pansexual* | 0.91 | 0.15 | 0.55 | | **0.72** | | **0.11** | **0.01** |
| *Queer* | 0.97 | 0.18 | 0.85 | | **0.73** | | **0.13** | **0.04** |
| *Asexual* | **0.66** | **0.15** | **0.02** | | **0.48** | | **0.11** | **< 0.01** |
| *Other sexual orientations ^b^* | **0.70** | **0.14** | **0.03** | | 0.76 | | 0.14 | 0.08 |
| **Caregiver education level** | **0.92** | **0.03** | **0.02** | | 1.05 | | 0.03 | 0.18 |

*Note.* *OR* = odds ratio; *S.E.* = Standard Error. Ref = Reference Group. Significant predictors (estimates *p* < .05) are bolded for emphasis. ^a^ Other gender identities include but are not limited to questioning, agender, xenogender, transfeminine, and transmasculine. ^b^ Other sexual orientations include but are not limited to heterosexual, questioning, abrosexual, demisexual, and omnisexual.
